# Diagnostic testing managed by hematopathology specialty and other laboratories: costs and patient diagnostic outcomes

**DOI:** 10.1186/1472-6890-14-17

**Published:** 2014-04-27

**Authors:** Nicole M Engel-Nitz, Benjamin Eckert, Rui Song, Priyanka Koka, Erin M Hulbert, Jeffrey McPheeters, April Teitelbaum

**Affiliations:** 1 Optum, Eden Prairie, MN, USA; 2 Novartis Molecular Diagnostics, Cambridge, MA, USA; 3 Present address Metamark Genetics, Cambridge, MA, USA; 4 Present address Heme Onc Associates, Carlsbad, CA, USA; 5 AHT BioPharma Advisory Services, Carlsbad, CA, USA; 6 Health Economics and Outcomes Research, Optum, 12125 Technology Drive, Eden Prairie, MN 53344, USA

**Keywords:** Diagnostic laboratory tests, Diagnostic costs, Hematopathology, Leukemia, Lymphoma

## Abstract

**Background:**

Successful management of patients with hematologic malignancies depends upon accurate and timely diagnosis, which frequently requires integration and interpretation of multiple tests. Our retrospective analysis compared diagnostic uncertainty, resource utilization, and costs for patients with diagnostic bone marrow (BM) tests managed by commercial laboratories.

**Methods:**

Patients with BM biopsies and suspected hematologic cancer/condition were identified from claims (2005–2011) within a large US health plan (coverage ≥6 pre- and ≥3-months post-biopsy). Cohorts defined by laboratories performing BM morphologic assessment/directing testing sequence: Genoptix (GX, specialty hematology-testing laboratory), large commercial laboratories (LL), other laboratories (OL). One-year post-biopsy changes in diagnosis or treatments, tests performed, and diagnostic/treatment medical costs (measured as per-patient-per-month [PPPM]) were examined.

**Results:**

The study population included 1,387 GX, 4,162 LL, and 19,115 OL patients with suspected hematologic malignancy/disease and BM morphology assessment. GX had lower diagnostic uncertainty measured between 2 time periods by diagnostic stability (no conditions the same; 6.16% GX, 8.04% LL, 9.73% OL; p < 0.001) and changes (≥1 condition different; 7.88% GX, 11.19% LL, and 14.08% OL; p < 0.001), fewer repeat BM biopsies, and fewer chemotherapy changes (30-days and 60-days post-initiation). One-year PPPM costs adjusted for patient characteristics differences were $8,202 GX, $7,711 LL, and $10,302 OL (p < 0.05); adjusted PPPM costs (excluding testing period) were $6,019 GX, $6,649 LL, and $7,801 OL (p < 0.05).

**Conclusions:**

Our data suggests that a hematopathology specialty laboratory may result in earlier final diagnosis, fewer subsequent diagnosis changes, reduced need for follow-on testing requiring repeat biopsy procedures, and may result in lower downstream healthcare costs. Further evaluations using medical chart abstractions or registries will be valuable.

## Background

Successful and optimal management of patients with hematologic malignancies depends upon early and accurate diagnosis. The establishment of a differential diagnosis may be challenging. Hematologic malignancies often have overlapping clinical presentations and the clinician is tasked with ruling out other malignant or non-malignant conditions before arriving at a final diagnosis [1]. Furthermore, accurate determination of stage and prognosis requires multiple testing platforms to discern cell types, cell lineage, degree of maturation or point of maturation arrest, and mutational and genetic/molecular status information [2,3]. Given the complexities of diagnosis, the heterogeneity of phenotypes, and variety of treatment options, [4,5] clinicians must consider histologic subtype and more and more importantly, molecular profile, as well as disease stage and other prognostic factors when planning appropriate treatment of hematologic malignancies [6].

Seeking to improve diagnostic accuracy for hematologic cancers, updated diagnostic guidelines have incorporated advances in diagnostic testing, including molecular profiling [6-8]. These advances have both improved diagnostic accuracy for hematologic cancers and increased the need for integration and interpretation of data from multiple tests often performed across multiple laboratory providers. The complexity of diagnosing hematologic malignancies has intensified with development of sophisticated laboratory tests as new molecular markers are identified. These markers are used to diagnose disease subtypes, as well as prognostic risk which impact treatment selection and disease monitoring [9,10].

Clinicians face challenges in keeping current with developments in diagnosis and treatment of hematologic malignancies [11,12]. For example, community-based oncologists may not see a large volume of patients with particular hematologic malignancies. When test material is referred for expert secondary hematopathology review, previously collected samples and test results may be interpreted differently and additional biopsies or tests may be requested [13]. Supplemental biopsies (bone marrow or others) may be needed to obtain a definitive diagnoses; this increases health care costs and often affects patient quality of life. In a study of Medicare patients, the estimated average direct costs of a bone marrow biopsy/aspirate for patients with chronic lymphocytic leukemia (CLL) was $1,722 (2007 USD) [14]. In the general population of patients with suspected lymphoma, average costs of a lymph node biopsy have been estimated at $822 (2005 USD) for a core needle to $3529 for an excisional biopsy [15]. The initial BM sample may provide enough tissue for basic diagnostic and prognostic tests. However, any additional tests may require one or more additional BM samples to complete, particularly when subsequent tests are not performed in the same laboratories. Thus, these secondary expert hematopathology reviews may increase healthcare costs, [14,15], as well as result in clinically meaningful diagnostic revision in up to 20% of the cases. Discrepancy rates vary with 10% for Hodgkin lymphoma and 75% for Burkitt’s lymphoma [13,16-18].

Clinicians are concerned about obtaining the useful and appropriate information in a timely and understandable manner. In a traditional diagnostic workflow, the skill and expertise of the individual completing the laboratory requisition forms determines ordering of appropriate tests. Differences in clinician awareness of updated diagnostic technology information may result in inadequate or inconsistent test ordering [12]. Subsequently, clinicians may need to make additional testing requests. Lack of diagnostic experience in a particular clinic[19] coupled with variable clinical presentations [20-23] may delay referral to a specialist or completion of a diagnostic workup. As diagnostic technologies evolve, differences in clinician awareness of updated information may lead to inconsistent test ordering and variable interpretation of test results [9,10,24].

To reduce the need for secondary review and improve diagnostic accuracy, a specialized hematopathology testing laboratory, Genoptix, designed a diagnostic workflow that addresses the main concerns associated with diagnostic testing in the community oncology setting: tests ordered, sampling errors, and interpretation/integration errors. This workflow places the hematopathologist in the role of central administrator and reviewer of each test throughout the course of case management. The course of testing is adjusted as test results become available and sampling errors can be quickly identified during integration of various testing results if an inconsistency arises. Results are correlated and interpreted until reaching a full diagnostic assessment. This workflow differs from that in a traditional community setting in which specimens are commonly distributed to disparate laboratories for analysis and the clinician performs the determination of tests and integrates the results from each laboratory report.

Comparisons between various laboratories and their workflow, as well as associated costs have not been well examined. To evaluate the impact of a hematopathology specialty laboratory on real-world patient population, we conducted a retrospective study comparing diagnostic changes, patterns of additional testing, treatment decisions, and health care costs for patients with suspected hematologic malignancies/conditions whose diagnostic tests were managed by specialty hematology laboratory and other commercial laboratories.

## Methods

**Figure 1:**
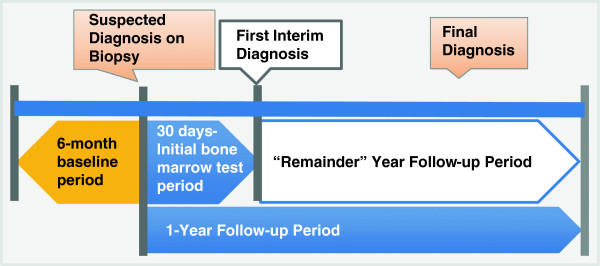
Study design.

This study used administrative claims data from the Optum Research Database (a proprietary database including claims from the large US health plan affiliated with Optum). Medical and pharmacy claims were retrospectively evaluated to identify the patients that had a bone marrow procedure (biopsy/aspirate) claim with suspected hematologic cancer/disease (*index date*; Figure 1) from 01 July 2005 through 30 June 2011 (eligibility period). The study further identified patients with diagnoses of MDS, myeloproliferative neoplasm (MPN), CLL, non-Hodgkin lymphoma (NHL), MM, other hematologic cancers, and other non-cancer hematologic conditions.

Health plan enrollment for a 6-month baseline period prior to the index date was required to assess patient characteristics and diagnostic/treatment history. Laboratory tests during the 30-days post-bone marrow biopsy were identified and patients followed for up to 1 year post-index. Diagnoses of hematologic cancers and conditions were identified in the medical claims. The *initial interim diagnosis* was identified based on the date of the first non-laboratory claim with a diagnosis of hematologic cancer/disease in the primary position at least 3 days after and <1 year post-index date. The first appearance of diagnoses for all hematologic cancers and conditions was identified over the follow-up period using these criteria, and the *final diagnosis* was the last such hematologic diagnosis identified.

Patient cohorts were assigned based upon the laboratories performing the bone marrow morphology assessment (directing the testing sequence): Genoptix (GX, a specialty hematology-testing laboratory), large commercial laboratories (LL), and other laboratories (OL) such as community hospital laboratories. Academic laboratories that sponsor hematopathology fellowships were excluded since these settings are likely to have a higher percentage of referral cases.

Diagnostic uncertainty following the initial diagnostic workup was estimated using 2 definitions comparing hematologic diagnoses between the initial interim and final diagnoses (up to three were retained on each of the initial and final diagnosis dates). Stability of diagnosis was defined as having at least 1 hematologic condition that was the same between the two time points; change in diagnosis was defined as having at least 1 condition that was different between the two intervals. The algorithm, pre-defined by protocol, explicitly excluded codes suggesting disease progression or hematologic signs/symptoms as instability or change. Other outcomes evaluated included the following: the number of tests performed, repeat bone marrow studies, time to final diagnosis, changes in chemotherapy in the 60-days post-biopsy, and testing costs and all-cause health care costs in the 1-year and 11-months remaining in the follow-up period.

Analysis was conducted using SAS version 9.2 (SAS Institute, Inc., Cary, NC, USA) for analytic dataset construction, descriptive statistics, and logistic regression; while Stata SE version 11 (StataCorp LP, CollegeStation, TX, USA) was used for other multiple regression analyses. Baseline and outcome variables were descriptively analyzed and per-patient-per-month (PPPM) costs were used to account for variable length of time observation. Multiple regression analysis were conducted using generalized linear models (GLM) with log link for total all-cause costs (1-year and remainder of year after the initial 30-day testing period), with this method selected due to the skewed distribution of the cost data. Logistic regression was used to assess repeat bone marrow biopsies, changes in chemotherapy, and diagnostic uncertainty (stability and change in diagnosis). Cox proportional hazard regression was used to model time to final diagnosis. The multiple regression analyses adjusted for patient characteristics and disease type [25-27].

## Results

The initial laboratory population consisted of 34,904 patients with suspected diagnosis of the entities of interest (Table 1). Patients with non-hematologic cancer and any other non-hematologic condition listed as the diagnoses on their bone marrow biopsy claims were removed from the final study population. Cohorts varied in the distribution of the suspected hematologic condition listed on bone marrow biopsy claims. The GX population had a higher percentage of patients with a non-malignant hematologic diagnosis (eg, anemia, thrombocytopenia) on the bone marrow biopsy claim compared to the other cohorts; the OL population had a higher percentage of patients with other hematologic cancer diagnosis.

**Table 1:** Patient suspected diagnosis, demographics, and baseline characteristics

		**Total**		**Genoptix**		**Large Labs**		**Other Controls**		**p-value**
**Study population size**		**(N = 24,664)**		**(N = 1,387)**		**(N = 4,162)**		**(N = 19,115)**		
		**n**	**%**	**n**	**%**	**n**	**%**	**n**	**%**	
Suspected diagnoses	MDS	859	3.48	23	1.66	128	3.08	708	3.70	<0.001
	MPN	1,004	4.07	61	4.40	159	3.82	784	4.10	0.578
	CLL	816	3.31	38	2.74	126	3.03	652	3.41	0.217
	MM	1,507	6.11	68	4.90	198	4.76	1,241	6.49	<0.001
	NHL	4,894	19.84	215	15.50	814	19.56	3,865	20.22	<0.001
	Other hematologic cancer	2,787	11.30	53	3.82	253	6.08	2,481	12.98	<0.001
	Other hematologic conditions	13,815	56.01	932	67.20	2,537	60.96	10,346	54.13	<0.001
Gender	Male	12,979	52.62	687	49.53	2,199	52.84	10,093	52.80	0.060
	Female	11,685	47.38	700	50.47	1,963	47.16	9,022	47.20	0.060
Age group	0-17	872	3.54	0	0.00	20	0.48	852	4.46	<0.001
	18-44	3,813	15.46	226	16.29	681	16.36	2,906	15.20	0.117
	45-64	10,272	41.65	636	45.85	1,911	45.92	7,725	40.41	<0.001
	65+	9,707	39.36	525	37.85	1,550	37.24	7,632	39.93	0.003
Insurance type	Commercial	18,639	75.57	1,068	77.00	3,280	78.81	14,291	74.76	<0.001
	Medicare	6,025	24.43	319	23.00	882	21.19	4,824	25.24	<0.001
Baseline therapy	Pre-index chemotherapy	1,908	7.74	83	5.98	291	6.99	1,534	8.03	0.003
	Pre-index radiation therapy	1,556	6.31	70	5.05	240	5.77	1,246	6.52	0.027
	History of radiation therapy	71	0.29	2	0.14	8	0.19	61	0.32	0.226
Geographical distribution	Northeast	2,391	9.69	62	4.47	512	12.30	1,817	9.51	<0.001
	Midwest	6,945	28.16	104	7.50	667	16.03	6,174	32.30	<0.001
	South	11,882	48.18	1,027	74.04	2,660	63.91	8,195	42.87	<0.001
	West and other	3,442	13.96	193	13.91	322	7.74	2,927	15.31	<0.001
	Unknown	4	0.02	1	0.07	1	0.02	2	0.01	0.200
Presence of down’s syndrome		31	0.13	1	0.07	1	0.02	29	0.15	0.092
Age (in years)	Mean (SD)	58.49	(18.04)	59.88	(15.12)	59.39	(15.46)	58.19	(18.73)	<0.001
	Median	60.00		60.00		60.00		60.00		
Pre-index Quan-Charlson comorbidity score	Mean (SD)	2.18	(2.11)	2.08	(2.11)	2.04	(2.01)	2.22	(2.13)	<0.001
	Median	2.00		2.00		2.00		2.00		
Distance to MSA	Mean (SD)	3.09	(11.73)	2.96	(10.26)	2.25	(10.26)	3.28	(12.11)	<0.001
	Median	0.00		0.00		0.00		0.00		

*Abbreviations*: *MDS* Myelodysplastic syndrome, *MPN* Myeloproliferative neoplasms, *CLL* Chronic lymphoid leukemia, *MM* Multiple myeloma, *NHL* Non-Hodgkin’s lymphoma, *MSA* Metropolitan statistical area, *SD* Standard deviation.

### Demographics, and baseline characteristics

The final study population (Table 1) with suspected hematologic malignancy/disease and bone marrow morphology assessment included 1,387 GX, 4,162 LL, and 19,115 OL patients. A patient may have had more than 1 suspected diagnosis. Compared to GX or LL patients, OL patients were slightly younger (average age 58.19 OL vs. 59.88 GX, 59.39 LL; p < 0.001) and overall approximately a quarter were enrolled in Medicare Advantage plans (25.24% OL, vs. 23.00% GX and 21.19% LL, p < 0.001).

Some differences in geographic distribution across cohorts were noted, with patients in the GX cohort more likely to be located in the South. Compared to the GX or LL cohorts, the OL cohort was slightly more likely to have had chemotherapy or radiation treatment during the baseline period.

### Diagnostic characteristics

Patients in the GX cohort were more likely to undergo more complex diagnostic tests during the initial 30-day testing period, most notably for cytogenetics/FISH (95.96% GX, 80.78% LL, and 51.68% OL) and molecular diagnostics (26.03% in GX, 14.27% in LL, and 9.31% in OL). Patients in the OL cohort were less likely to have these tests performed, and when done, were more likely to be performed at a different lab type.

**Figure 2:**
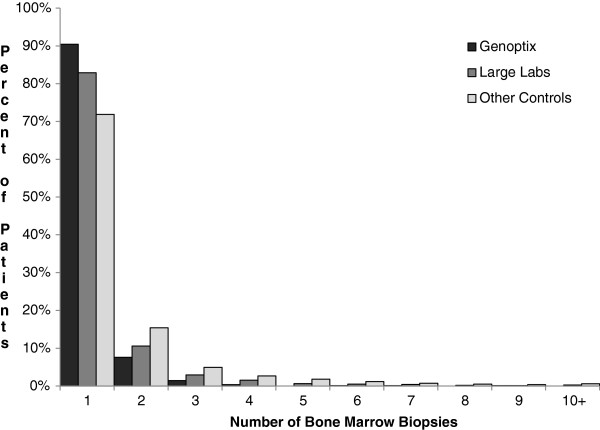
Distribution of bone marrow biopsies received per patient and by laboratory.

The number of tests (Table 2) varied across the 1-year follow-up period. The distribution of BM biopsies was skewed with the majority of patients receiving 1 BM (Figure 2). The LL cohort had the fewest total tests and the GX and OL cohorts appearing more similar (Table 2). The average time to final diagnosis differed across the cohorts, ranging from 36 days for GX to 41 days for OL (marginal difference, p = 0.051). The median time to final diagnosis was roughly 2 weeks, with the OL cohort having a shorter time by 2 days. The Cox proportional model hazard ratios (reference group OL cohort) of reaching a final diagnosis by any point in time within the initial 30-day testing period were 1.002 (p = 0.0029) for the GX cohort and 0.95 for the LL cohort (p = 0.0002). However, at any point in time during the post-30 day testing period, the GX cohort had a roughly 23% higher hazard than the OL cohort of having reached a final diagnosis by that point (HR = 1.23, p = 0.0007), and the LL cohort had a roughly 10% higher hazard of having reached a final diagnosis at any given point in time (HR = 1.10, p = 0.005). Substantially fewer GX patients underwent repeat marrow biopsies (Table 2; 9.59% GX, vs. 17.11% LL, and 28.16% OL, p < 0.001), with differences remaining after adjusting for type of hematologic malignancy diagnosed and other characteristics (OR: GX 0.31 [0.26, 0.37]; LL 0.56 [0.51, 0.62]).

**Table 2:** Diagnostic characteristics: test utilization, frequency of repeat bone marrow biopsy, and stability and change in diagnosis

			**Total**	**Genoptix**	**Large labs**	**Other controls**	**p-value**
**Study population size**			**(N = 24,664)**	**(N = 1,387)**	**(N = 4,162)**	**(N = 19,115)**	
**Diagnostic flow patterns - Test counts - Post-index fixed 1-year follow-up period (Full population)**	Number of bone marrow tests	Mean (SD)	10.92 (11.14)	17.79 (7.08)	3.79 (7.43)	11.97 (11.35)	<0.001
		Median	8.00	16.00	1.00	9.00	
	Number of cancer-related tests	Mean (SD)	9.00 (15.91)	5.41 (10.93)	7.54 (14.15)	9.57 (16.52)	<0.001
		Median	1.00	0	0	2.00	
	Number of hematology-related tests	Mean (SD)	2.00 (7.36)	2.00 (5.93)	2.00 (7.27)	1.00 (7.47)	<0.001
		Median	2.00	2.00	2.00	2.00	
	Number of all laboratory tests (includes all diagnostic tests in claims data)	Mean (SD)	25.48 (25.57)	28.37 (15.01)	17.03 (21.00)	27.11 (26.69)	<0.001
		Median	0.00	0.00	0.00	0.00	
**Repeat bone marrow biopsy**	Number of bone marrow biopsies	Mean (SD)	1.56 (1.42)	1.13 (0.49)	1.33 (1.04)	1.64 (1.52)	<0.001
		Median	1.00	1.00	1.00	1.00	
	Individuals with multiple bone marrow biopsies	n (%)	6,227 (25.25)	133 (9.59)	712 (17.11)	5,382 (28.16)	<0.001
		Odds ratio		0.307	0.563		
		Confidence interval		(0.255, 0.371)	(0.514, 0.617)		
		P-value		P < 0.001	P < 0.001		
		Logistic model adjusted for gender, age, region (South), Medicare, initial diagnosis (MM, MDS, CLL, other NHL, MPN, other hematologic cancer/conditions), and baseline Charlson comorbidity score.					
**Stability of diagnosis**	Stable	n (%)	20,333 (90.76)	1,203 (93.84)	3,500 (91.96)	15,630 (90.27)	<0.001
	Unstable	n (%)	2,069 (9.24)	79 (6.16)	306 (8.04)	1,684 (9.73)	<0.001
	Logistic model of having unstable diagnosis in follow-up	Odds ratio		0.866	0.992		
		Confidence interval		(0.68, 1.103 )	(0.867, 1.134)		
		P-value		P = 0.2427	P = 0.9014		
		Logistic model adjusted for gender, age, region (Northwest), Medicare, initial diagnosis (MM, CLL, other NHL, MPN, other hematologic cancer/conditions), baseline Charlson comorbidity score, baseline chemotherapy, baseline radiation therapy, baseline inpatient visit, and baseline number of bone marrow-related tests.					
**Change in diagnosis**	No change	n (%)	19,437 (86.76)	1,181 (92.12)	3,380 (88.81)	14,876 (85.92)	<0.001
	Change	n (%)	2,965 (13.24)	101 (7.88)	426 (11.19)	2,438 (14.08)	<0.001
	Logistic model of diagnosis change in follow-up	Odds ratio		0.824	0.939		
		Confidence interval		(0.722, 0.94)	(0.867, 1.018)		
		P-value		P = 0.0040	P = 0.1256		
		Logistic model adjusted for gender, age^2^, region (Northwest), initial diagnosis (MM, MDS, other NHL, MPN, other hematologic cancer/conditions), baseline Charlson comorbidity score, baseline chemotherapy, and baseline number of bone marrow-related tests.					

*Abbreviations*: *MDS* Myelodysplastic syndrome, *MPN* Myeloproliferative neoplasms, *CLL* Chronic lymphoid leukemia, *MM* Multiple myeloma, *NHL* Non-Hodgkin’s lymphoma, *SD* Standard deviation.

**Table 3:** Chemotherapy utilization and health care cost outcomes

			**Total**	**Genoptix**	**Large Labs**	**Other Controls**	**p-value**
**Study population size**			**(N = 24,664)**	**(N = 1,387)**	**(N = 4,162)**	**(N = 19,115)**	
**Changes to chemotherapy**	Within 30 days of starting chemotherapy	%		4.58%	6.68%	7.37%	0.91
	Within 31–60 days of starting chemotherapy	%		1.78%	3.68%	5.12%	0.001
	Adjusted odds ratio within 60 days of starting chemotherapy	Odds ratio		0.718	1.197		
		Confidence interval		0.472, 1.093	0.98, 1.463		
		P-value		0.1224	0.0784		
		Logistic model adjusted for age, region (Northwest, South), Medicare, initial diagnosis (MM, other NHL, other hematologic cancer/conditions), baseline Charlson comorbidity score, and baseline chemotherapy.					
**Testing paid cost PPPM - Post-index fixed 1-year Follow-up period**	Bone marrow biopsy costs	Mean (SD)	$1,804.34 (12,424.61)	$149.54 (1,006.24)	$736.74 (5,638.49)	$2,156.87 (13,842.72)	<0.001
	Bone marrow tests costs	Mean (SD)	$305.18 (596.83)	$1,130.03 (1,022.85)	$249.81 (383.29)	$257.39 (547.21)	<0.001
	Other cancer-related tests costs	Mean (SD)	$292.68 (897.24)	$109.31 (405.88)	$199.39 (653.65)	$326.30 (963.49)	<0.001
	Other hematological tests costs	Mean (SD)	$47.93 (258.88)	$20.97 (67.77)	$34.30 (184.72)	$52.85 (280.35)	<0.001
	Total testing costs	Mean (SD)	$2,449.16 (12,689.30)	$1,409.84 (1,674.85)	$1,214.87 (5,842.95)	$2,793.32 14,127.97)	<0.001
	Mean costs include individuals with zero costs						
**Mean healthcare costs PPPM during 1-year fixed follow-up period by Service type**	Pharmacy cost	Mean (SD)	$624.69 (1,309.22)	$571.42 (1,279.36)	$578.84 (1,214.31)	$638.53 (1,330.84)	0.008
	Medical costs	Mean (SD)	$8,556.06 (21,267.46)	$4,790.58 (9,286.12)	$5,830.33 (13,479.36)	$9,422.77 (23,117.31)	<0.001
	Ambulatory cost	Mean (SD)	$3,279.69 (5,288.34)	$2,042.87 (3,618.11)	$2,759.93 (4,467.36)	$3,482.60 (5,529.99)	<0.001
	Office costs	Mean (SD)	$1,370.38 (2,591.48)	$1,281.19 (2,468.00)	$1,516.33 (2,648.42)	$1,345.07 (2,586.69)	<0.001
	Outpatient cost	Mean (SD)	$1,909.31 (4,338.71)	$761.68 (2,157.14)	$1,243.60 (3,251.64)	$2,137.53 (4,626.67)	<0.001
	Emergency services cost	Mean (SD)	$54.10 (216.34)	$36.42 (120.02)	$40.09 (129.11)	$58.43 (235.87)	<0.001
	Inpatient cost	Mean (SD)	$4,742.55 (19,060.20)	$1,515.11 (7,315.71)	$2,664.49 (11,325.53)	$5,429.20 (20,851.55)	<0.001
	Other cost	Mean (SD)	$479.72 (2,746.48)	$1,196.18 (1,179.72)	$365.82 (1,200.40)	$452.54 (3,045.91)	<0.001
	Total cost	Mean (SD)	$9,180.74 (21,433.97)	$5,362.00 (9,492.76)	$6,409.17 (13,676.32)	$10,061.30 (23,281.54)	<0.001
**All-cause paid costs PPPM: Summary of multiple Regression model results**	***Paid costs-remainder year (excluding initial 30-day period)***		Adjusted Costs	$6,019	$6,649	$7,801	
	***Paid costs-1 year***		Adjusted Costs	$8,202	$7,711	$10,302	
	GLM model, adjusting for gender, age, age^2^, region, final diagnosis, baseline Charlson comorbidity score, baseline chemotherapy, baseline radiation therapy, baseline inpatient stay, baseline number of bone marrow-related tests.						

*Abbreviations*: *GLM* generalized linear models, *MM* Multiple myeloma, *NHL* Non-Hodgkin’s lymphoma, *PPPM* Per-Patient-Per-Month, *SD* Standard deviation.

Stability of initial diagnosis (Table 2) varied across cohorts (unstable diagnoses in 6.16% GX, 8.04% LL, and 9.73% OL; p < 0.001) with odds ratios (OR) of unstable diagnosis of 0.87 for GX [95% CI: 0.68, 1.10] and 0.99 for LL [95% CI: 0.87, 1.13].

GX patients had lower percentages of diagnosis changes (Table 2); OL patients had the highest (7.88% GX, 11.19% LL, and 14.08% OL; p < 0.001) with differences between GX and OL remaining after adjustments (OR: 0.82 for GX [95% CI: 0.72, 0.94] and 0.94 for LL [95% CI: 0.87, 1.02]).

### Utilization and costs

In the 30 day period after starting chemotherapy (Table 3), changes in treatment were noted: 4.58% GX, 6.68% LL, and 7.37% OL (p = 0.91) of patients changed chemotherapy (prevalence rate ratios for GX vs. OL: 0.5732, p = 0.013; for LL vs. OL: 0.8348, p = 0.115). Within 31 to 60 days of chemotherapy treatment initiation, GX patients had fewer changes in chemotherapy (an additional 1.78% GX, 3.68% LL, and 5.12% OL; p = 0.001; prevalence rate ratios for GX vs. OL: 0.3204, p < 0.001; for LL vs. OL: 0.6607, p = 0.005). Results between the cohorts were not statistically significant after multiple regression adjustment for differences in patient characteristics (OR: 0.72 for GX [95% CI: 0.47, 1.09] and 1.20 for LL [95% CI: 0.98, 1.46]).

The distribution of health care cost was skewed. Unadjusted analyses indicated that GX cohort total healthcare costs ($5,362.00) were lower than costs for the LL ($6,409.17) and OL ($10,061.30) cohorts (1-year, PPPM). Unadjusted costs were lower for GX in all service categories with the exception of the ‘Other’ category, which includes lab services. PPPM costs for testing over the 1-year follow-up were highest for the OL cohort ($2,793), followed by GX ($1,410) and LL ($1,215), p < 0.001.

The 1-year PPPM costs adjusted for differences in patient characteristics were $8,202 GX, $7,711 LL, and $10,302 OL p < 0.05). Adjusted costs PPPM excluding the initial 30-day testing period were $6,019 GX, $6,649 LL, and $7,801 OL (p < 0.05). The cost models found that some interactions terms between laboratory cohort and disease type were statistically significant. However, the results were in the same direction for all disease types. Thus, the adjusted results shown for each cohort are based on these models and shown for the average population.

## Discussion

This retrospective study examined diagnostic patterns and diagnostic, clinical and economic outcomes for patients with suspected hematologic cancers/conditions. Overall, the diagnostic outcomes examined in the study generally favored Genoptix relative to the OL cohort, with fewer changes in diagnosis, fewer repeat bone biopsies or changes in chemotherapy treatments. Differences between the Genoptix and the LL groups were smaller and were not compared directly.

Population characteristics of the Genoptix and LL cohorts differed from the OL, including initial suspected diagnosis listed on the bone marrow biopsy. Multiple regression analyses adjusted for final or suspected diagnoses as appropriate, but the differences in diagnoses may reflect not only underlying differences in the actual diseases of the patient populations, but also differences in coding practices across institutions. However, other differences in severity or complexity of the patients’ conditions may not be adequately reflected in claims data.

The distribution of test types differed for Genoptix compared with the other cohorts. A higher percentage of Genoptix patients were more likely to have undergone complex diagnostic tests and Genoptix was consistently recorded as the provider on the biopsy-related tests during the 30-day testing period. For the OL, the complex diagnostic tests were more likely to have been performed at a lab type other than the one providing bone marrow morphology assessment, requiring clinicians and pathologists using the other labs to integrate test results from multiple sources.

The multiple regression analyses suggested that there may be an advantage for Genoptix over other lab types in reaching a final diagnosis earlier. There was also an advantage for LL compared with the OL in this regard, but the effect for Genoptix was slightly more than double that of the LL. However, the difference in mean time to diagnosis was only a few days in the unadjusted numbers. The clinical impact of a difference that a few days make on the diagnosis is unknown; yet, the earlier the diagnosis is made the more rapidly the patient can be assigned to an appropriate treatment plan. The difference between mean and median time to diagnosis indicates there was a large amount of variability in the time to diagnosis, suggesting that even if on average the difference was a few days, there was a subset of patients who were subject to much lengthier delays in diagnosis. Since the definitions for identifying final diagnosis were created independently of the cohorts we do not expect that any effects due to specific definitions used would vary across cohorts other than the variation due to the initial and final diagnoses.

The Genoptix cohort had the lowest rates of unstable diagnosis and unexpected changes in final diagnosis, followed by the LL and then the OL cohorts. This finding parallels the hypothesis that improvements in diagnostic certainty and completeness occurs with initial hematopathology specialty laboratory assessment. Claims data may not adequately capture this type of diagnostic instability and the algorithm developed in the study may not reflect actual changes in diagnoses. However, the results of the study are consistent with the ranges reported in the literature, particularly with an NCCN study that identified a 6% discordance rate in diagnoses for B-cell NHL [13].

The changes in chemotherapy treatments favored the Genoptix cohort in descriptive statistics; however, the relationship was not statistically significant in multiple regression analyses after adjusting for potential confounding variables in the model. This may also be due to small size in number of patients that experienced a change in chemotherapy. Since the relationship between the laboratory testing and specific clinical outcomes is unclear, these results should be interpreted with caution.

Results for cost and utilization suggest some differences between the cohorts. The source of costs differed across the cohorts, with the ‘other’ (i.e. laboratory) services being the biggest driver for Genoptix costs compared with hospitalization costs for the OL cohort. The adjusted analyses suggested that overall, paid costs for the GX cohort were lower than for the OL cohort. The LL cohort was not the analytic reference group and was not compared directly to the Genoptix cohort. In general, cost differences between the Genoptix and LL groups were either small, or costs were slightly lower for the LL group.

Overall, our results support that compared to the OL for diagnosis of hematologic malignancies and MDS, a hematopathology specialty laboratory may result in more rapid final diagnosis, fewer changes in diagnoses, reduction in need for follow-on testing including repeat biopsy procedures, and may result in lower overall health care costs. Additional research will be needed to confirm whether the use of a hematopathology specialty laboratory minimizes potential harm from misdiagnosis compared to other types of labs and whether there is an efficiency benefit in using a specialty laboratory compared to large laboratories.

### Limitations

Certain limitations are associated with using claims data for research. Reasons for laboratory tests and other clinical parameters are not readily apparent in claims data. Presence of a diagnosis code on a medical claim is not proof positive of the disease; the disease may have been coded incorrectly. For some hematologic malignancies, the ICD-9-CM coding schematic does not distinguish between disease subsets and the heterogeneity and diversity of the conditions. The study did not evaluate quality of life as this type of data is not available in claims data. Due to the overall survival differences between the various hematologic diseases and too few patients for a specific diagnosis, follow-up times were not considered adequate to assess survival in this study.

While this study used multiple regression analyses to adjust for differences in patient populations between the laboratory cohorts, there may have been differences that could not be identified. Clinician characteristics, such as varying degrees of expertise in recognizing these conditions, may have impacted outcomes as well. Selection bias may account for where biopsies were sent for evaluation: more complicated or confusing cases may have been sent to a specialty laboratory initially. In other instances, contractual considerations and insurance coverage may have determined laboratory selection. In addition, it is not clear if a repeat bone marrow biopsy was necessitated by an initial inadequate sample (i.e., poor technical quality).

Differences in treatment patterns cannot be evaluated as appropriate care since reasons for those treatment patterns were not available. Similarly, while the study developed an algorithm to identify progression and change in diagnoses in claims data, the algorithm cannot be verified based upon claims data alone. This study also excluded academic centers with hematopathology fellowships, as the patients seen in academic centers could differ significantly in their underlying disease in ways unlikely to be measurable in claims data. Furthermore, claims data do not contain quality of life and disease severity information. Thus, it is unclear if the differences observed in this study would also be observed in clinical outcomes.

## Conclusions

Stability and changes in hematologic diagnoses varied by the type of lab performing the initial testing on the bone marrow sample, with a trend for fewer changes observed for the hematology specialty lab. Repeat bone marrow biopsies, changes in chemotherapy, and costs in the period following initial diagnostic workup were lower for patients whose samples were assessed by a specialty laboratory versus other laboratory types (after adjusting for patient population differences).

Further exploration of alternative diagnostic testing approaches and their impact on costs and clinical outcomes, as well as the impact of management by specialized (hematopathologist) as compared to general-pathology services on outcomes is warranted. Validation of these findings through medical chart abstractions or registries will be important in illuminating the impact of hematopathology specialty services on patient outcomes.

## Competing interests

Funding for this study was provided by Novartis Molecular Diagnostics to Optum. Five (NEN, RS, PK, EMH, and JM) of the 7 authors are employed by Optum. One author (BE) was formerly employed by Novartis during the course of the research and is now with Metamark Genetics. One author (AT) was formerly employed by Optum during the course of the research and is now with Heme Onc Associates and AHT BioPharma Advisory Services. All authors have read and approved this manuscript.

## Authors’ contributions

NEN designed the study, conducted the study, and drafted the manuscript. BE conceived the study, participated in the study design, approved the protocol and variables analysed, and drafted the manuscript. RS, PK, EMH, and JM identified the data and performed the statistical analysis and revised the manuscript for critically important intellectual content. AT participated in the study design, interpretation of study results, and revised the manuscript for critically important intellectual content. All authors read and approved the final manuscript.

## Pre-publication history

The pre-publication history for this paper can be accessed here:

http://www.biomedcentral.com/1472-6890/14/17/prepub
